# Novel small non-coding RNAs of Epstein-Barr virus upregulated upon lytic reactivation aid in viral genomic replication and virion production

**DOI:** 10.1128/mbio.04060-24

**Published:** 2025-04-08

**Authors:** Sagarika Banerjee, Dipayan Bose, Steve Johnson, Jie Liu, Herbert Virgin, Erle S. Robertson

**Affiliations:** 1 Departments of Otorhinolaryngology-Head and Neck Surgery, Perelman School of Medicine at the University of Pennsylvania, Philadelphia, Pennsylvania, USA; 2 Department of Pathology, Washington University School of Medicine in St. Louis, St. Louis, Missouri, USA; Princeton University, Princeton, New Jersey, USA

**Keywords:** Epstein-Bar virus, non-coding RNA, lytic reactivation, capture hybridization analysis of RNA targets, apoptosis, OriLyt

## Abstract

Epstein-Barr virus (EBV) employs various strategies for long-term survival, including the expression of non-coding RNAs (ncRNAs). This study uncovers and characterizes two novel EBV-encoded ncRNAs, p7 and p8, which are upregulated during lytic reactivation and interact with both viral and host genomes. These ncRNAs bind to cellular RNA transcripts, significantly reducing ARMCX3 mRNA levels, while p8 also influences PTPN6 and RPL24 expressions. Although p7 does not directly bind to LMP1 RNA but both ncRNAs found to downregulate LMP1 expression. Furthermore, these ncRNAs interact with the OriLyt region of EBV genome, promoting viral DNA replication. Functional assays indicate that p7 and p8 enhance cell proliferation and inhibit apoptosis by modulating the p53 pathway and suppressing pro-apoptotic proteins. These findings highlight the role of p7 and p8 in supporting EBV persistence by regulating viral replication, cell survival, and immune evasion, making them promising targets for therapeutic strategies in EBV-related diseases.

**IMPORTANCE:**

Epstein-Barr virus (EBV) employs diverse strategies for long-term persistence in the host, including the expression of viral non-coding RNAs (ncRNAs) that manipulate key cellular pathways to promote viral replication and immune evasion. This study identifies two novel EBV-encoded ncRNAs, p7 and p8, which are upregulated during lytic reactivation and interact with both viral and host genes to regulate viral DNA replication and promote host cellular survival. By modulating apoptotic and proliferative pathways, p7 and p8 facilitate viral reactivation while promoting host cell survival, highlighting their potential as critical regulators in EBV-driven oncogenesis. This discovery expands our understanding of EBV-host interactions, suggesting p7 and p8 as targets for novel therapeutic strategies in EBV-associated malignancies.

## INTRODUCTION

Epstein-Barr virus (EBV) is the first human virus that has been shown to be associated with cancer etiologically (1). EBV is an omnipresent human gammaherpesvirus that exists in over 90% of adults around the world. The virus initially infects epithelial cells and B cells in the oral pharynx and establishes latency in the cell compartment of memory B cells (2). In most cases, the virus co-exists with the host without any severe pathophysiological condition, but a small population of latently infected individuals may develop lymphocytic and epithelial malignancies such as Burkitt’s lymphoma, Hodgkin’s lymphoma, T-cell lymphoma, nasopharyngeal carcinoma (NPC), and gastric cancer (3, 4). In the latently infected cells, only 11 of approximately 95 viral open reading frames are expressed (5, 6). Moreover, EBV is also reported to encode more than 30 non-coding RNAs (ncRNA) (7). The transcriptome of the EBV is complex and consists of many alternatively spliced transcripts resulting in different gene expressions. For EBV, three different latency programs have been reported to date, which are characterized by various patterns of coding and non-coding of viral gene expression.

Non-coding RNAs (ncRNAs) play critical roles in cellular functions and are categorized arbitrarily as either short ncRNAs (<200 nucleotides) or long ncRNAs (>200 nucleotides) (8). Long non-coding RNAs (lncRNAs) are currently defined as transcripts of approximately 200 nt or more, but without open reading frames (ORFs) (9). Several studies revealed that lncRNAs have regulatory roles, including regulation of apoptosis and invasion, reprogramming of induced pluripotent stem cells, cell fate markers, and parental imprinting (10, 11). MicroRNAs (miRNAs) belong to a specific type of short ncRNAs and are responsible for gene regulation. These miRNAs are approximately 22 nt in length and negatively regulate gene expression either by inducing mRNA degradation or by repressing translation in mammalian cells (12–15). EBV-transformed lymphoblastoid B-cell lines (LCL cells) and the nasopharyngeal cells have revealed 25 EBV precursor miRNAs (pre-miRNAs), from which nearly 40 mature miRNAs are processed (7, 16). The EBV miRNAs are grouped into two clusters within the EBV genome: one in the BART gene intronic regions (miR-BART1 to miR-BART-20) and the other in the BHRF1 gene’s untranslated regions (UTRs) (miR-BHRF1-1 to miR-BHRF1-3) (17, 18). Interestingly, in latently infected epithelial cells, miR-BARTs were found to be abundantly expressed (19, 20), but at a significantly lower rate in B cells, suggesting their role in epithelial carcinogenesis. In addition, miR-BARTs have also been shown to be strongly expressed in gastric carcinoma cell (EBV-GC) lines and tissues infected with EBV (21), in which only a few viral genes are constitutively expressed.

In this study, we discovered two novel small ncRNAs of EBV that are highly expressed in lytic-reactivated LCL1 cells. RNA-seq analysis of total RNA from reactivated LCL1 cells revealed two discrete peaks of about 50 reads each at 140 kb genomic position on the positive strand that do not match to any known gene annotations. One peak was at 139,553 bp (peak 7 or p7), and the other was at 139,817 bp (peak 8 or p8) on the EBV AG876 strain, next to the BILF2 gene. Genome-wide location analysis of p7 and p8 using Capture hybridization analysis of RNA targets (CHART) clearly suggested that p7 and p8 not only promote viral replication by binding to the OriLyt region and increase virion productivity but these two ncRNAs were also found to hinder LMP1 expression and expression of certain host genes, which ultimately lead to the completion of efficient viral lytic life cycle and inhibit the host cell apoptosis.

## RESULTS

### Detection of the two ncRNAs in lytic-reactivated LCLs

**Fig 1:**
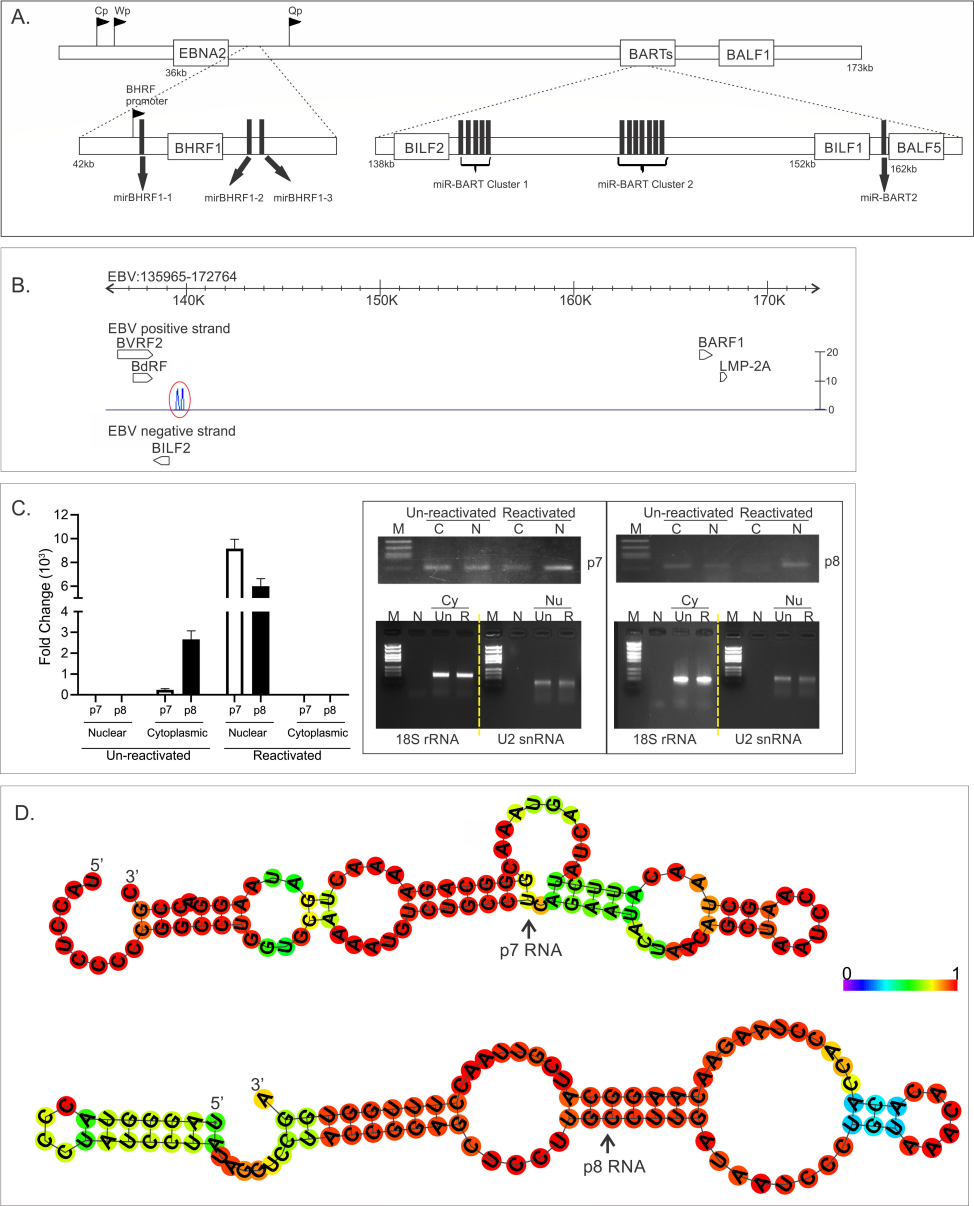
Genomic position and secondary structures of the novel small RNAs. (A) Schematic representation of the location of EBV ncRNAs within the EBV genome. EBV miRNAs are derived from mainly EBNA1, BHRF1, and BART transcripts. The BART miRNAs form two clusters within the BART introns, with the exception of mirBART2, which lies further downstream between the BILF1 and the BALF5 genes. (B) RNA-seq of the total RNA extracted from reactivated LCL1 yielded two novel small transcripts (encircled) at ~140 kbp position next to BILF2. (C) Sub-cellular localization of p7 and p8 RNA in un-reactivated and reactivated LCL1 cells. qRT-PCR was done using the extracted RNAs from nuclear and cytoplasmic fractions of un-reactivated and reactivated (5dpi) LCL1 cells. Fold change of the RNAs (cytoplasmic and nuclear) using 18S rRNA and U2 snRNA as endogenous controls for cytoplasmic and nuclear RNA, respectively, was calculated to determine the subcellular localization of the small RNAs. (D). Secondary structure based on MFE, of p7 and p8 RNAs predicted by RNA-fold software. The structure below is colored by base-pairing probabilities. For unpaired regions, the color denotes the probability of being unpaired.

**Fig 2:**
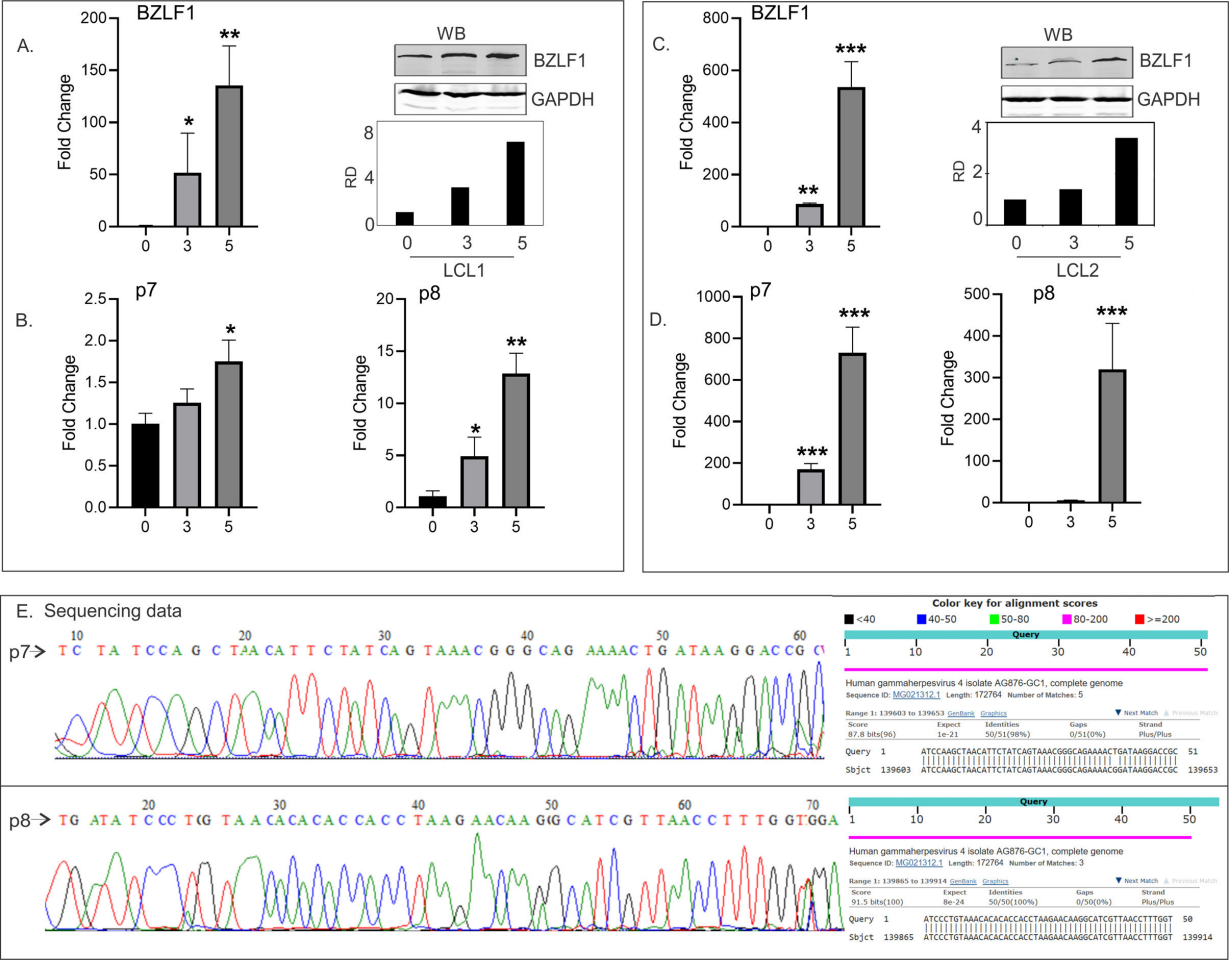
Expression of p7 and p8 RNAs in lytic reactivated LCLs. (A and C) BZLF1 expression in reactivated LCL1 (A) and LCL2 (C) cells by qRT-PCR and western blot. RNA extracted from un-reactivated and reactivated LCL1 and LCL2 cells 3 and 5 dpi were reverse transcribed, and qPCR was done on the cDNA to look for the expression of BZLF1. GAPDH/18S rRNA was used as the endogenous control. Expression of un-reactivated cells (LCL,0) was taken as 1. (B and D) p7 and p8 RNA expressions in LCL1 (B) and LCL2 (D) cells were detected by qRT-PCR. RNA extracted from BJAB cells (BJ), unreactivated (0), and reactivated LCL1 and LCL2 cells at 3 and 5 dpi were reverse transcribed, and qPCR was done on the cDNA. GAPDH/18S rRNA was used as the endogenous control. Expression of un-reactivated cells (LCL, 0) was taken as 1. (E) Electropherogram of Sanger sequencing for p7 and p8 amplicons, along with their respective BLAST results are shown. Total RNA was extracted from the LCL1 cells 5 dpi, and RT-PCR was done to amplify the p7 and p8 cDNAs. The amplicons were gel-extracted and subjected to Sanger sequencing for identification.

Upon RNA-seq analysis of total RNA from 3-day post-reactivated (TPA + BA) LCL1 cells, we observed two discrete peaks of about 50 reads each at 140 kb genomic position on the positive strand that does not match any known gene annotations. One peak was at 139,553 bp (peak 7 or p7), and the other was at 139,817 bp (peak 8 or p8) on the EBV AG876 strain (accession no. DQ279927), next to the BILF2 gene (Fig. 1A and B). These 100 nt transcripts showed multiple predicted stem-loop structures (Fig. 1D), not typical of pre-miRNAs. Furthermore, miRNA predictor software tool was used, which confirmed them not to be pre-miRNAs. We performed a qRT-PCR on the total nuclear and cytoplasmic RNA of unreactivated and reactivated (3 dpi) LCL1 cells, and we found that the two small RNAs are detected mostly in the cytoplasm of un-reactivated LCL1 cells, but upon lytic reactivation, they are mostly localized in the nucleus (Fig. 1C). We also reactivated two clonal LCL cell lines (LCL1 and LCL2) and compared the transcript level in un-reactivated and reactivated cells at 3 and 5 days post-reactivation. We found that although both p7 and p8 ncRNAs were detected in un-reactivated LCLs, they were detected at a 6-fold to 10-fold higher amount upon lytic reactivation (5 dpi) in both LCL1 and LCL2 cells (Fig. 2B and D). Successful lytic reactivation was confirmed by an increased BZLF1 transcript and protein expression (Fig. 2A and C), with BZLF1 being an early lytic gene of EBV. To confirm that the amplified product of the specific PCRs was in fact the transcript sequence, we performed a reverse transcription followed by PCR using total RNA extracted from reactivated LCL1 cells, gel purified the amplification product of 70 bp, and sent it for Sanger sequencing. The electropherogram of the sequences is shown in Fig. 2E (left panel), and the sequences were subjected to BLAST to confirm the identity of the transcripts (Fig. 2E, right panel). Apart from the lymphoblastoid cell lines, we also detected p7 and p8 ncRNA in reactivated and reactivated nasopharyngeal carcinoma cells (NPCs) transformed by M81 strain of EBV (data not shown). We detected a very high induction of p7 ncRNA and an 8-fold higher amount of p8 ncRNAs upon lytic reactivation (3 dpi). Because of the unusually high level of replication by M81 strain in NPCs, we did detect high amount of p7 and p8 ncRNA transcripts, and both ncRNAs could be detected in un-reactivated M81 cells. We thus confirmed the identity of the ncRNAs in M81 cell lines by PCR and Sanger sequencing. These data thus confirm the presence of two previously uncharacterized EBV-encoded non-coding RNAs (p7 and p8) whose expression was found to increase during lytic reactivation.

### Determining the potential targets of these ncRNAs will lead to the understanding of their role

**Fig 3:**
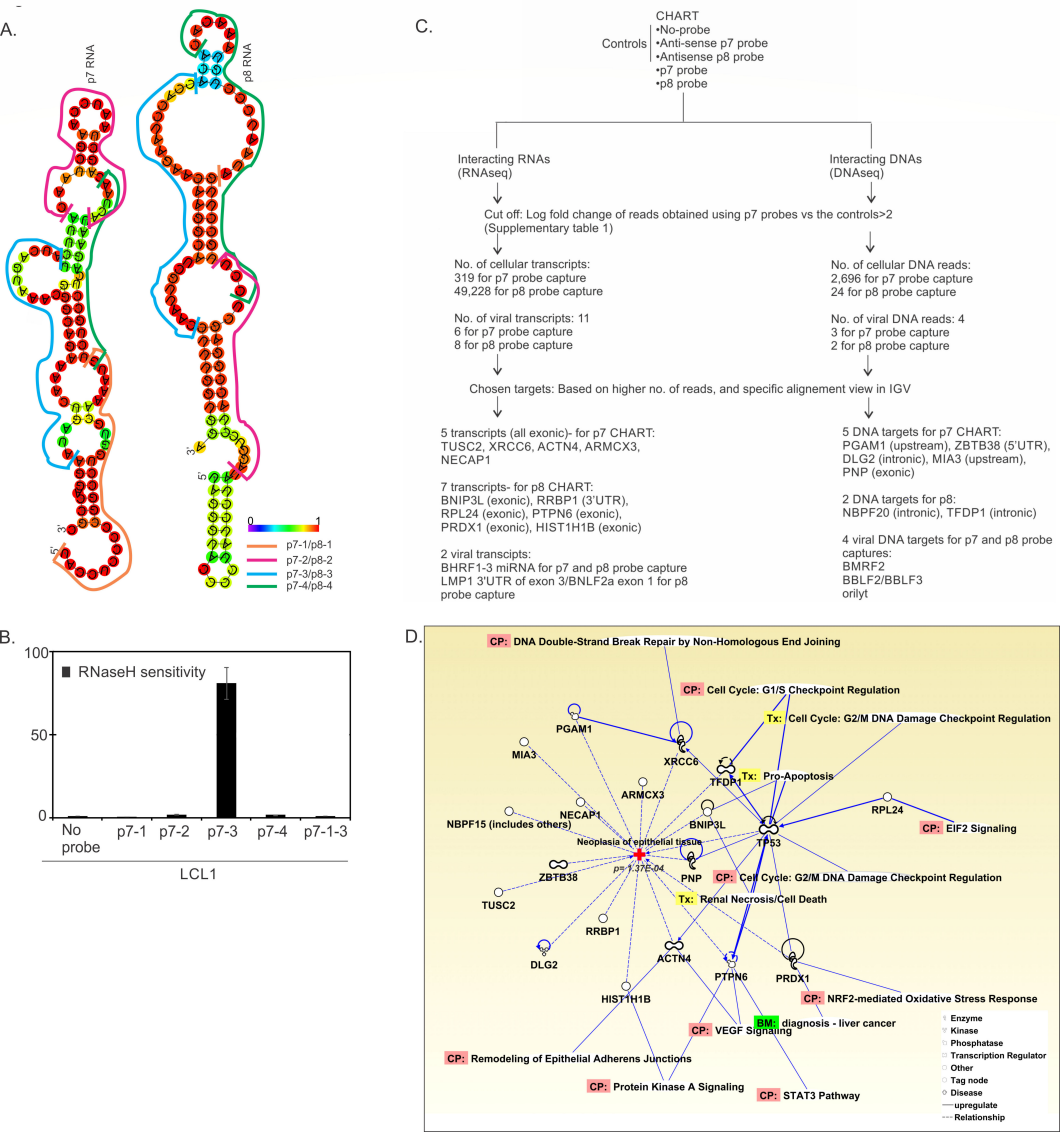
Cellular and viral DNA/RNA-binding sites of p7 and p8 RNAs determined by CHART. A hybridization-based strategy, CHART, uses complimentary oligonucleotides to purify the RNAs together with its targets from reversibly cross-linked extracts. (A) Position of the binding sites for the capture probes (p7-1, p7-2, p7-3, and p7-4 for p7 capture) used for RNase H sensitivity assay. (B) Analysis of RNase H sensitivity on the binding of p7 and p8 probes to the accessible sites on p7/p8 RNAs. RNase H sensitivity represents the ratio of cleaved to un-cleaved RNA. (C) Workflow of CHART analysis. CHART was performed with reactivated (3 dpi) LCL1 cells, using p7-3 and p8-3 probes to pull out DNA/RNA bound to p7 and p8 RNAs. Bound DNA and RNA were quantitated and determined by DNA-seq and RNA-seq, respectively. (D) Ingenuity pathway analysis (IPA) shows the predicted DNA/RNA targets of p7 and p8 RNAs to significantly correlate to neoplasia of epithelial cells, either directly, or indirectly via modulation of p53.

Sub-cellular localization of the ncRNAs generally gives an idea about their possible role (22), and finding these ncRNAs enriched in the nucleus post-lytic reactivation is suggestive of a possible nuclear role. In order to find the viral and cellular gene targets of these ncRNAs, we performed capture hybridization analysis of RNA targets (CHART) using biotinylated complementary oligo-deoxyribonucleotides (DNA probes) that binds to the accessible region the ncRNAs and capture the ncRNA bound together with the targets that may include RNA, DNA, and/or protein from cross-linked chromatin (23, 24). To find such capture probes for these two ncRNAs, we first performed an RNase H sensitivity assay, where the formation of DNA (capture probe)-ncRNA hybrids (Table S1) at the accessible region in the ncRNAs induces cleavage by RNase H, and then, the degree of RNase H sensitivity can be determined (25) by RT-qPCR using specific primers (Table S2) of the p7 and p8 ncRNAs (Fig. 3B). No amplification is suggestive of effective RNase H sensitivity of the capture probes. We designed three capture probes for each of the ncRNAs (Fig. 3A; Table S1) and selected only one capture probe for each of the ncRNAs to show RNase H sensitivity: p7-3 (probe designed from site 3 of p7 ncRNA) capture probe and p8-3 (probe designed from site 3 of p8 ncRNA) capture probe that showed 80% and 45% RNase H sensitivity, respectively (data not shown). Using the RNase H-sensitive capture probes, we performed CHART separately for p7 and p8 ncRNA. We have performed duplicate captures with the selected p7 capture probes (designated in the capture sequence alignment figures as p7-1 and p8-1 captures), and a single capture experiment with the selected p8 probe (designated in the capture sequence alignment figures as p8 captures). We included a “no probe” negative control and “antisense capture oligo deoxyribonucleotides” of each of the capture probes (anti-p7-3 and anti-p8-3) as negative controls for the CHART assay (Tables 1 to 6; Table S1). The biotinylated capture probe-pulled down targets such as RNA, DNA, or protein that was identified by RNA-seq, DNA-seq, and mass spectrometry, respectively and identified potential RNA, DNA, and protein targets of the p7 and p8 ncRNAs (Fig. 3C; Table S3). To identify the top hits in the DNA-seq and RNA-seq data, we first included a cutoff criterion of log fold change >2 for reads obtained using capture probes compared with the negative controls, and a significant *P*-value (<0.05) for the comparison. Using the cutoff, we identified 319 host cellular and six viral targets as potential RNA targets for p7 ncRNA, and 49,228 host cellular and eight viral targets as potential RNA targets for p8 ncRNA (Table S3; Fig. 3C). For the potential DNA targets, using the same cutoff, we identified 2,696 cellular gene targets and three viral targets for p7 ncRNA, whereas only 24 cellular gene targets and two viral targets for p8 ncRNA (Table S3; Fig. 3C). From this list of possible targets, we narrowed down to the top hits based on higher number of reads and specific alignment views. The top hit RNA targets included the exonic region of 5 cellular transcripts for p7 ncRNA and the exonic or 3′UTR region 7 cellular transcripts for p8 ncRNA and 1–2 viral transcripts (Table S3; Fig. 3C). The top DNA targets included the upstream region, 5′UTR region, intronic and exonic region of 5 cellular gene targets for p7, and intronic region of 2 cellular DNA targets for p8 (Table S3; Fig. 3C), as well as four viral DNA targets for both p7 and p8 ncRNAs (Table S3; Fig. 3C). These cellular DNA and RNA targets that we identified as top hits have significant (*P* = 1.37E−04) connection, either directly or indirectly (through p53) to the development of neoplasia of epithelial cells as seen by Ingenuity Pathway Analysis (Fig. 3D).

**TABLE 1:** Selected p7 cellular RNA target from RNA-seq data of CHART experiment

Controls			Test			p7 *P* value	Adj p7 *P* value	p7 logFC	Target region	Gene	Human chr coordinates	Role of target genes
No-probe reads	Antisense p7 reads	Antisense p8 reads	p7_1 reads	p7_2 reads	p8 reads							
0	0	0	210	158	19	0.044	0.543	7.533	All of exon 2, start of exon 3	TUSC2	chr3:50363001–50364000	Tumor suppressor
0	0	0	90.1	71.6	5.9	0.036	0.543	6.355	Exon 12	XRCC6	chr22:42059001–42060000	DNA repair gene
0	0	0	52.2	39.3	0	0.044	0.543	5.546	Exon 1	ACTN4	chr19:39138001–39139000	Transcriptional coactivator in cancer, cancer biomarker, helps in metastasis
0	0	0	21	26.1	19	0.033	0.543	4.617	Exon 5	ARMCX3	chrX:100880001–100881000	Senescence biomarker
0	0	0	18.8	19.2	0	0.003	0.543	4.320	Exon 5, 7	NECAP1	chr12:8248001–8249000	Role not sure

**TABLE 2:** Selected p8 RNA cellular target from RNA-seq data of CHART experiment

Controls			Test			p8 logFC	Target region	Gene	Human chr coordinates	Role of target genes
No-probe reads	Antisense p7 reads	Antisense p8 reads	p7_1 reads	p7_2 reads	p8 reads					
0	0	0	0	0	248.8	7.964	Exon 6	BNIP3L	chr8:26268001–26269000	Induces apoptosis
0	0	0	0	0	178.8	7.490	3'UTR of exon 2	RRBP1	chr20:17640001–17641000	Promotes cancer progression
0	0	0	0	0	151	7.247	Exon 2, 3, 4, 5, 6	RPL24	chr3:101404001–101405000	Required for cancer cell growth
0	0	0	0	0	145.1	7.190	Exon 2, 3, 4, 5, 6	RPL24	chr3:101401001–101402000	Required for cancer cell growth
0	0	0	0	0	131.6	7.050	Exon 3, 5, 6, 7, 8, 9, 10, 11, 12, 13, 14, 15, 16	PTPN6	chr12:7069001–7070000	Suppressor of Mcl-1, Bcl-2, and survivin
0	0	0	0	0	127.4	7.003	Exon 2, 3, 4, 5, 6 and 3'UTR of exon 4	PRDX1	chr1:45980001–45981000	Tumor suppressor
0	0	0	0	6.593	124	6.965	Exonic	HIST1H1B	chr6:27835001–27836000	Regulator of gene transcription through chromatin remodeling, nucleosome spacing, and DNA methylation

**TABLE 3:** Selected p8 RNA viral target from RNA-seq data of CHART experiment

Controls			Test			EBV coordinates	Target genes
No-probe reads	Antisense p7 reads	Antisense p8 reads	p7_1 reads	p7_2 reads	p8 reads		
0	0	0	0	0	26.988	chrEBV:167001–168000	LMP1 3'UTR of exon 3/BNLF2a exon 1
0	0	0	0	5.394	7.590	chrEBV:41001–42000	BHRF1-3

**TABLE 4:** Selected p7 cellular DNA targets from DNA-seq data of CHART experiment

Controls			Test			p7 *P*-value	Adj p7 *P*-value	p7 logFC	Target region	Gene	Human chr coordinates	Role of target genes
No-probe reads	Antisense p7 reads	Antisense p8 reads	p7_1 reads	p7_2 reads	p8 reads							
0	0	0	9.702	10.699	0	0.016	0.287	3.485	Upstream; downstream	PGAM1; EXOSC1	chr10:99185001–99186000	PGAM1 overexpression aids in cancer cell proliferation
0	0	0	8.797	11.033	2.173	0.036	0.287	3.448	UTR5	ZBTB38	chr3:141088001–141089000	Transcriptional repressor ZBTB38 negatively regulates transcription and levels of the MCM10 replication factor on chromatin, leading to DNA damage
0	0	0	6.985	8.0243	0.836	0.022	0.287	3.088	Intronic	DLG2	chr11:83649001–83650000	Tumor suppressor
0	0	0	6.339	7.6899	0.669	0.031	0.287	3.003	Upstream	MIA3	chr1:222790001–222791000	Tumor suppressor
0	0	0	7.115	6.3525	0.167	0.018	0.287	2.951	Exonic	PNP	chr14:20937001–20938000	High PNP in cancer

**TABLE 5:** Selected p8 cellular DNA target from DNA-seq data of CHART experiment

Controls			Test			p8 logFC	Target region	Gene	Human chr coordinates	Role of target genes
No-probe reads	Antisense p7 reads	Antisense p8 reads	p7_1 reads	p7_2 reads	p8 reads					
0	0	0	5.045	2.675	7.356	3.063	Intronic	NBPF20, NBPF9, SEC22B	chr1:145107001–145108000	Altered expression is associated with several types of cancer.
0	0	0	0.776	1.672	7.356	3.063	Intronic	NBPF20, NBPF9, SEC22B	chr1:145108001–145109000	
0	0	0	3.363	2.675	6.855	2.974	Intronic	NBPF20, NBPF9	chr1:145085001–145086000	
0	0	0	0.647	3.678	6.52	2.911	Intronic	NBPF20, NBPF9, SEC22B	chr1:145101001–145102000	
0	0	0	0.906	2.006	5.517	2.704	Intronic	NBPF20, NBPF9, PDE4DIP	chr1:145035001–145036000	
0	0	0	7.115	3.343	5.016	2.589	Intronic	NBPF20, NBPF9	chr1:145082001–145083000	
0	0	0	1.682	0.669	4.514	2.463	Intronic	NBPF20, NBPF9, PDE4DIP	chr1:145011001–145012000	
0	0	0	2.199	3.678	4.18	2.373	Intronic	NBPF20, NBPF9, PDE4DIP	chr1:145027001–145028000	
0	0	0	3.363	2.675	4.012	2.326	Intronic	NBPF20, NBPF9, PDE4DIP	chr1:145069001–145070000	
0	0	0	4.14	4.346	3.678	2.226	Intronic	NBPF20, NBPF9, PDE4DIP	chr1:145029001–145030000	
0	0	0	0.906	1.337	3.678	2.226	Intronic	NBPF20, NBPF9, PDE4DIP	chr1:145042001–145043000	
0	0	0	0.906	1.337	3.678	2.226	Intronic	NBPF10, NBPF20	chr1:145402001–145403000	
0	0	0	1.035	1.003	3.511	2.173	Intronic	NBPF20	chr1:144531001–144532000	
0	0	0	3.363	6.353	3.511	2.173	Intronic	NBPF20, NBPF9, PDE4DIP	chr1:145025001–145026000	
0	0	0	3.493	21.73	3.511	2.173	Intronic	NBPF20, NBPF9, PDE4DIP	chr1:145065001–145066000	
0	0	0	0.776	1.672	3.344	2.119	Intergenic	FAM72C (dist = 105,841), NBPF20 (dist = 126,811)	chr1:144019001–144020000	
0	0	0	7.115	3.009	3.344	2.119	Intronic	NBPF20, NBPF9, PDE4DIP	chr1:144941001–144942000	
0	0	0	1.294	2.006	3.344	2.119	Intronic	NBPF20, NBPF9, PDE4DIP	chr1:145063001–145064000	
0	0	0	4.398	3.343	3.177	2.062	Intronic	NBPF20, NBPF9, PDE4DIP	chr1:145024001–145025000	
0	0	0	2.458	2.675	3.177	2.062	Intronic	NBPF20, NBPF9, SEC22B	chr1:145110001–145111000	
0	0	0	3.105	1.337	3.009	2.003	Intronic	NBPF20, NBPF9	chr1:145094001–145095000	
0	0	0	0	0.669	8.025	3.174	Intronic	TFDP1	chr13:114278001–114279000	TFDP1 is a transcription factor that plays a role in cell cycle regulation, apoptosis, and senescence.
0	0	0	0	0	6.186	2.845	Intronic	TFDP1	chr13:114279001–114280000	
0	0	0	0.388	0	3.009	2.003	Intronic	TFDP1	chr13:114281001–114282000	

**TABLE 6:** Selected p7 and p8 RNA viral target from DNA-seq data of CHART experiment

Controls			Test			EBV coordinates (NC_007605)	Target genes
No-probe reads	Antisense p7 reads	Antisense p8 reads	p7_1 reads	p7_2 reads	p8 reads		
0	0	0	1.034887	5.683855	2.674988	171001–171823	Terminal repeats
0	0	0	3.363382	17.05156	2.674988	151001–152000	LMP2
0	0	0	3.8800826	70.88101	7.857778	40301–41293	orilyt

Mass spectroscopic analysis of the p7 and p8 targets identified TRIP12, a E3 ubiquitin protein ligase as an interacting partner for both p7 and p8, whereas SMG1 specifically for only p7 (data not shown). To properly ascertain the roles of p7 and p8 on the functionality of these protein, extensive study needs to be performed. This study used CHART to identify nuclear roles of EBV ncRNAs p7 and p8 by capturing their DNA, RNA, and protein targets. RNA-seq and DNA-seq analyses revealed many cellular and viral targets, with top hits related to genes involved in epithelial neoplasia. Mass spectrometry further identified TRIP12 as an interacting partner for both ncRNAs, and SMG1 specifically for p7, suggesting functional implications that warrant further investigation.

### Validation of potential DNA and RNA targets of the ncRNAs

**Fig 4:**
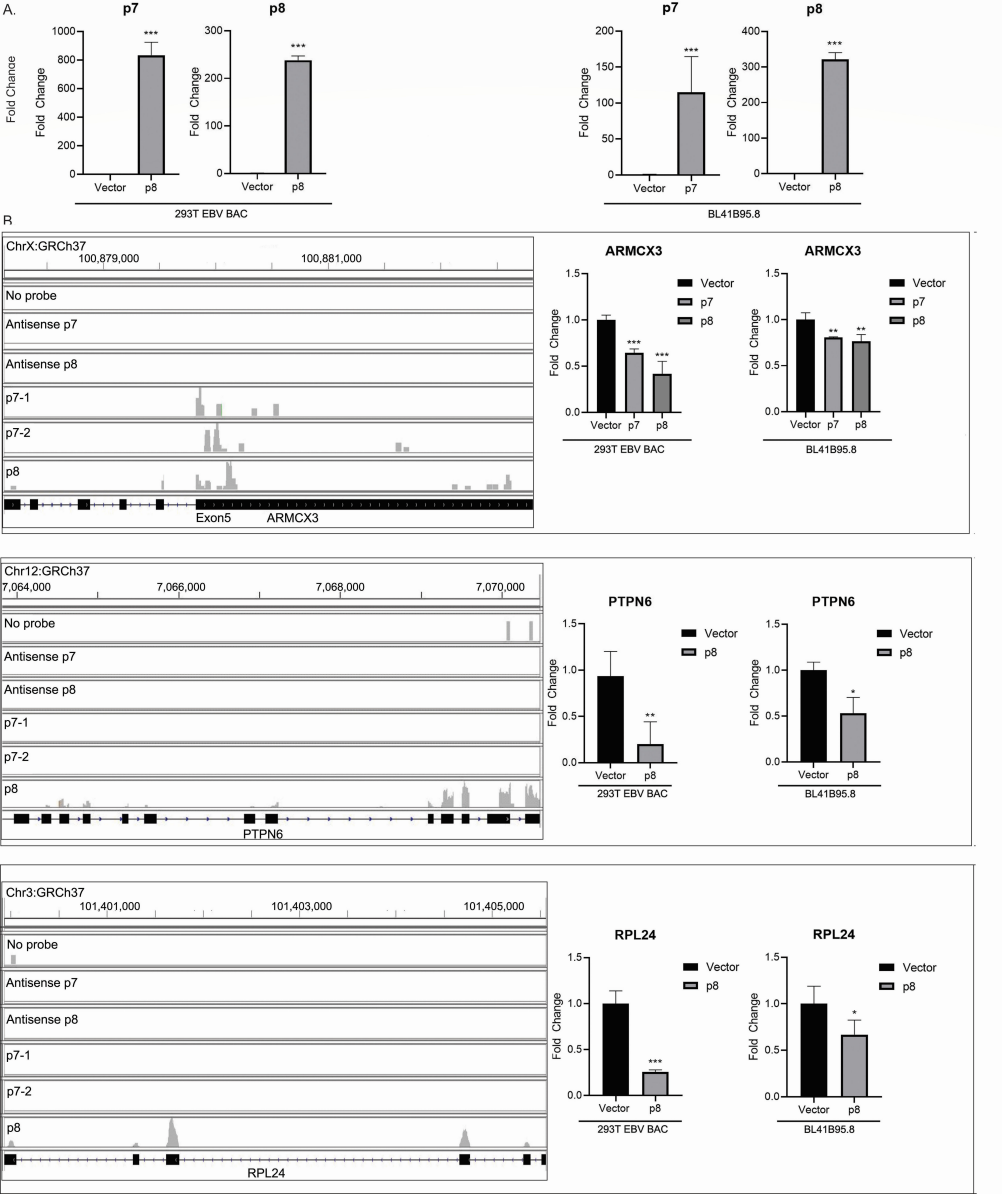
Validation of cellular RNA targets of p7 and p8 RNAs. For the validation of the predicted cellular RNA targets of p7 and/or, p8 RNAs, 293T EBV GFP cell line and BL41-B95.8 cell line were used. Both of these cell lines are transformed with EBV B95.8 strain of the virus, which has a deletion in the location of the p7/p8 transcript. 293T EBV GFP cell line and BL41-B95.8 cell line were transfected with the p7 or, p8 transcript cloned into pcDNA3.1 expression vector or, with empty vector, and the target gene suppression upon ectopic expression of the p7/p8 RNAs were checked by real time reverse transcription PCR. (A) The expression of p7 and p8 RNA was determined by RT-PCR, 72 h post-transfection. Fold change was calculated by taking the expression of the RNAs in empty vector transfection as 1. GAPDH and 18srRNA were used as the endogenous control. (B) Cellular target gene suppression upon ectopic expression of the p7 and/or p8 RNAs. Left panel, shows the alignment of the RNA-seq data in IGV, for each of the targets. Negative controls comprising a capture with no-probe, antisense of p7 probe, and antisense of p8 probe are shown in separate tracks. The p7 capture was repeated twice (p7-1 and p7-2), and the alignments are shown in separate tracks, along with a separate p8 capture track. The genomic locations are mentioned. Right panel shows the target gene suppressions upon ectopic expressions of the p7/p8 RNAs, validating the binding sites observed by RNA-seq alignments of the capture sequencing in the left panel.

**Fig 5:**
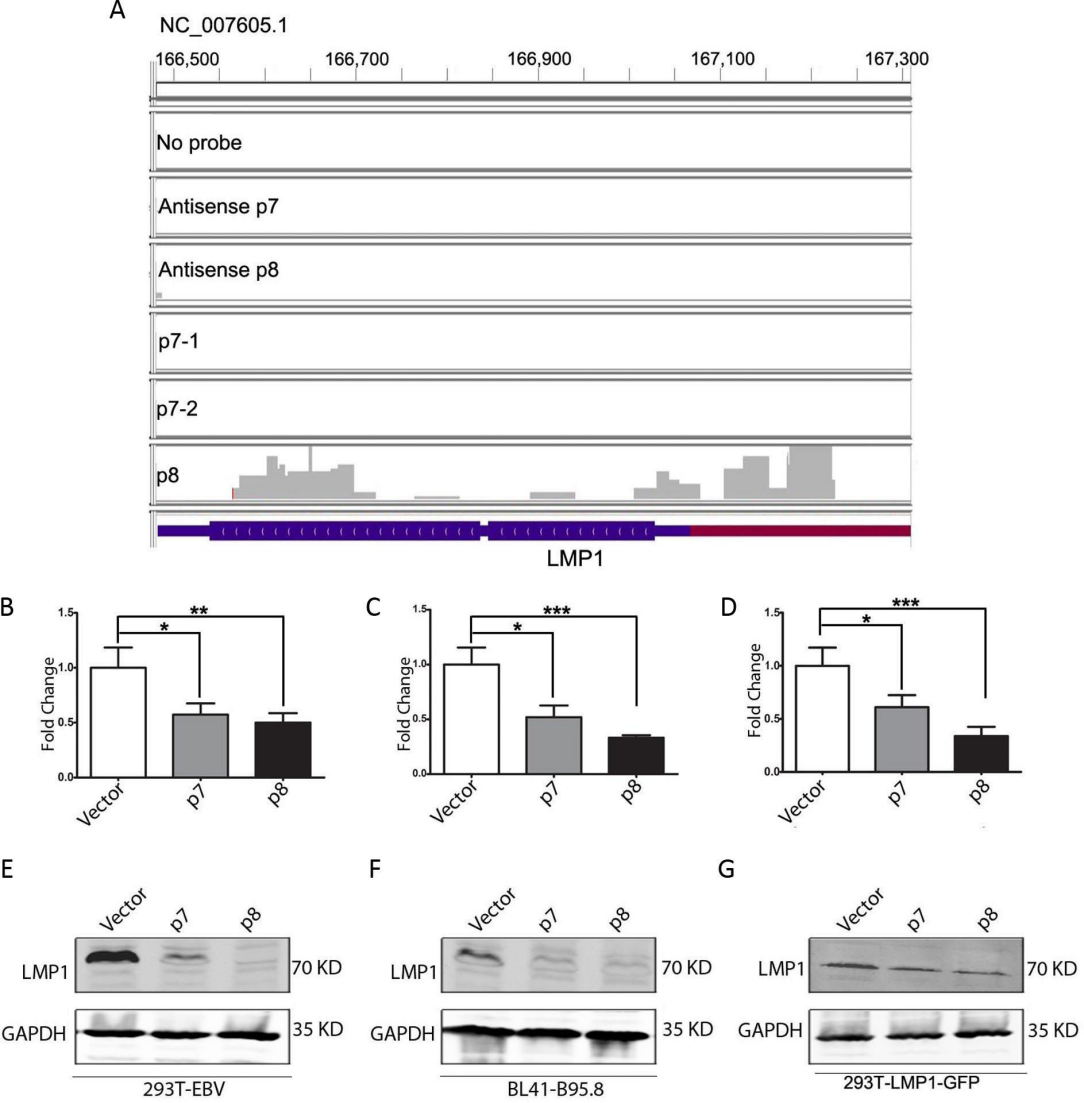
Validation of viral RNA target of p7 and p8 RNAs. For the validation of the predicted viral RNA target (LMP1) of p7 and/or, p8 RNAs, 293T EBV GFP cell line, and BL41-B95.8 cell line were used. Both of these cell lines are transformed with EBV B95.8 strain of the virus, which has a deletion in the location of the p7/p8 transcript. 293T EBV GFP and BL41-B95.8 cell lines were transfected with the p7 or, p8 transcript cloned into pcDNA3.1 expression vector or, with empty vector. (A) The IGV alignment of the RNA-seq data, to show the binding site of the p8RNA to be LMP1 exonic and 3’UTR region. Negative controls comprising a capture with no-probe, antisense of p7 probe, and antisense of p8 probe are shown in separate tracks. The p7 capture was repeated twice (p7-1 and p7-2), and the alignments are shown in separate tracks, along with a separate p8 capture track. The genomic locations are mentioned in the IGV alignment. (B–G) The LMP1 gene suppression upon ectopic expression of the p7/p8 RNAs was checked by real-time reverse transcription PCR and western blot.

In order to validate the potential cellular and viral DNA and RNA targets of the ncRNAs, we ectopically expressed the ncRNAs in an EBV-transformed cell line (BL41-B95-8) that lack the p7 and p8 ncRNA-encoding region (26) and checked the expression level of the target genes in presence and absence of the ncRNAs. We chose HEK293T (epithelial) and BL41 (B cell) stable cell lines transfected by BAC-EBV tagged with GFP. The EBV genome in this BAC construct is obtained from B95-8 strain that has a 12 kb deletion, including the region that spans the p7 and p8 ncRNAs (26) (Fig. 4A). We observed a change in the expression of some of the target genes upon ectopic expression of the ncRNAs (Fig. 4 and 5). ARMCX3 gene expression was found to be significantly suppressed upon ectopic expression of the ncRNAs in both 293T-EBV-GFP and BL41-B95-8 cell lines (Fig. 4B, right panel). The alignment of the reads from duplicate p7 captures and p8 capture with the ARMCX3 gene human reference sequence (GRCh37.p13) shows the binding sites of the ncRNAs to exonic regions of the gene (Fig. 4B, left panel). We also detected suppression in the PTPN6 gene expression in both the cell lines upon ectopic expression of p8 ncRNA (Fig. 4B, right panel), which matched with the alignment of the capture reads to the PTPN6 gene sequence showing p8 binding sites in the PTPN6 exonic regions (Fig. 4B, left panel). There was significant suppression in RPL24 gene expression upon ectopic p8 ncRNA expression (Fig. 4B, right panel), which again matched with the alignment of the capture reads to the RPL24 gene that showed p8 ncRNA-binding sites at the exonic regions of the gene (Fig. 4B, left panel). The expression of the EBV latent protein LMP1 was found to be significantly suppressed in 293T EBV-GFP and BL41-B95-8 cells upon ectopic expression of the p8 ncRNAs (Fig. 5B through G) in both RNA and protein levels as evident from real-time PCR and western blot data. In fact, EBV-negative 293 cells transfected with the LMP1 expression vector (LMP1-AcGFP) and the ncRNA expression vectors showed suppression of LMP1 at the transcript and protein levels (western blot) (Fig. 5E through G). The alignment of the capture reads with the LMP1 gene shows the binding of the p8 ncRNA to the LMP1 transcript end and 3’UTR region (Fig. 5A).

These findings suggest regulatory role of p7 and p8 in both host and viral gene expression at RNA and protein levels.

### Enhancement of viral replication in the presence of ncRNAs

EBV, during reactivation, initiates its lytic replication from some specific regions on the chromosome known as OriLyt, which spans between 40,300 and 41,300 bp region of EBV genome (NC_007605.1). Previous reports suggest that OriLyt requires a DNA-RNA hybrid to initiate the replication cycle (27). Different proteins, like Zta, stabilize this RNA-DNA hybrid structure and tether other proteins that are required for the DNA replication to initiate.

**Fig 6:**
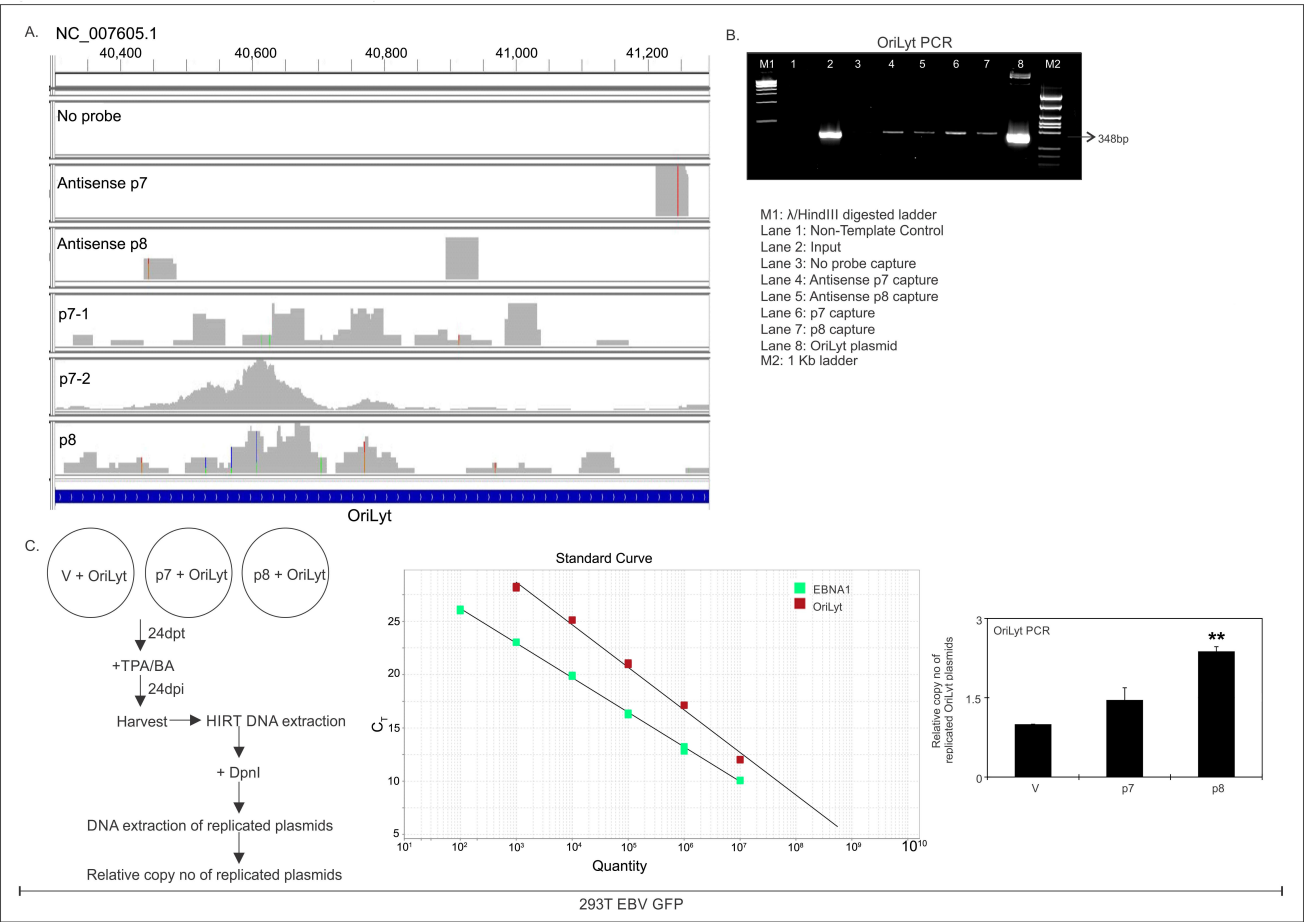
The p7 and p8 RNAs bind to OriLyt DNA and enhance replication. (A) IGV alignment of the DNA-seq data, to show the binding site of the p8RNA to be OriLyt region of EBV. Negative controls comprising a capture with no-probe, antisense of p7 probe, and antisense of p8 probe are shown in separate tracks. The p7 capture was repeated twice (p7-1 and p7-2), and the alignments are shown in separate tracks, along with a separate p8 capture track. The genomic locations are mentioned in the IGV alignment. (B) The pull-down DNA by the capture, part of which was sent for DNA-seq was subjected to PCR using OriLyt primers from the predicted binding site to validate the IGV results. The PCR products were run on 1.5% agarose gel and stained with EtBr. (C) Replication assay was done to check if p7 and p8 RNA binding to OriLyt help in enhancing replication. 293T EBV GFP cells (transformed with B95.8 strain of EBV) were transfected with empty (v), or, p7, or, p8 expression plasmids, and OriLyt plasmid. Twenty-four hours post-transfection, the cells were treated with TPA/BA to reactivate EBV. Twenty-four hours post-reactivation, the cells were harvested, and HIRT DNA extraction was performed to get rid of the genomic DNA. DpnI treatment was done on the extracted DNA (comprised of OriLyt plasmid and viral genomic DNA), to degrade non-replicating viral DNA. The replicated OriLyt plasmid DNA was extracted post-DpnI treatment and used to calculate the relative copy number of replicated OriLyt plasmids. Copy no of OriLyt plasmid was normalized by the copy no. of EBNA1 DNA in each of the three conditions. The standard curve of OriLyt PCR and EBNA1 PCR are shown, and the relative copy no of OriLyt plasmid is represented graphically.

DNA sequencing results from the CHART analysis revealed OriLyt as one of the important EBV DNA targets for both p7 and p8. The p7 and p8 probe-captured DNA sequence were aligned with the EBV genome in IGV alignment tool. The alignment shows significant overlap with the OriLyt region (Fig. 6A). To confirm the presence of OriLyt DNA in the p7 and p8 CHART pull-down DNA, a PCR detection method was used, in which specific primers for OriLyt was used. In this PCR, 1 ng of OriLyt plasmid and a no-template control was included as the positive and negative control for PCR detection, respectively. EtBr staining clearly showed the expected amplicon size for the OriLyt-specific PCR (Fig. 6B). IGV alignment result of the DNA-seq also indicated binding of the antisense p7 and p8 probe with the OriLyt, and the PCR results also confirmed the IGV alignment results. A replication assay was performed to see if the p7 and p8 ncRNAs aid in enhanced replication. 293T-EBV GFP cells were co-transfected with p7, p8, or empty vector plasmid along with OriLyt plasmid and 24 h post-transfection, and the cells were reactivated with TPA and BA. After 24 h of reactivation, the cells were harvested, and HIRT DNA extraction was performed to get rid of the genomic DNA followed by Dpn1 treatment that degrades non-replicating viral DNA. The replicated OriLyt plasmid DNA was extracted, and the relative copy number of the replicated OriLyt plasmid was calculated and normalized by the copy number of the EBNA1 DNA. Standard curve for the OriLyt and the EBNA1 was prepared (Fig. 6D) and was used to calculate the copy number of the OriLyt in the experimental system. p7- and p8-transfected samples showed nearly 1.5 and 2.5 times increase in the OriLyt copy number, respectively, compared with that of the empty vector control. This finding further reconfirms the CHART results and shows the role of these two ncRNAs in viral DNA replication.

In conclusion, our findings highlight that the non-coding RNAs p7 and p8 bind to the OriLyt region of the EBV genome, playing a critical role in viral replication initiation. The CHART analysis, IGV alignment, and PCR validation demonstrate significant interaction between these ncRNAs and OriLyt. Furthermore, the replication assay reveals that p7 and p8 substantially enhance OriLyt replication, underlining their functional importance in EBV’s lytic cycle.

### Increased virus productivity in the presence of p7 and p8 RNA

**Fig 7:**
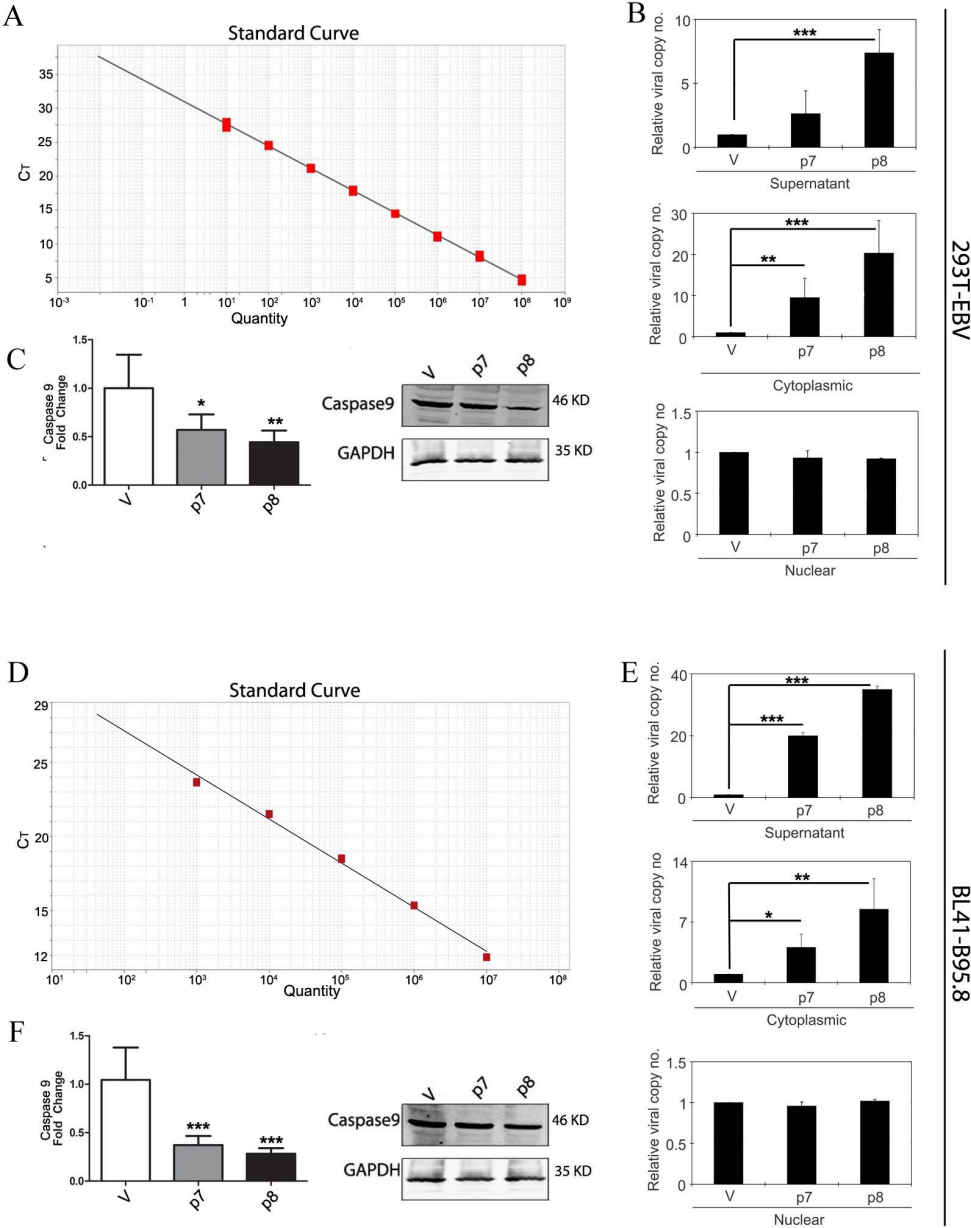
The p7 and p8 RNAs aid in enhanced viral productivity. 293T EBV GFP cell line and BL41-B95.8 cell line are transformed with EBV B95.8 strain of the virus which has a deletion in the location of the p7/p8 transcript. 293T EBV GFP cell line (A–C) and BL41-B95.8 cell line (D–F) were transfected with the p7 or, p8 transcript cloned into pcDNA3.1 expression vector or, with empty vector, and BZLF1 expression vector. The cell culture supernatant was collected, and the cells were harvested 5 days post-transfection. (B, E) From the cell culture supernatant, viral particles were pelleted by ultracentrifugation, and from the cells, cytoplasmic and nuclear fractions were separated. The pellet from the supernatant was subjected to DNase I treatment to get rid of any non-viral extraneous DNA. Viral DNA was extracted from the viral pellet, and cytoplasmic and nuclear DNA was extracted from the transfected cells. Real-time PCR for EBNA1 was done to quantitate the copy no of viral DNA in the cell culture supernatant in the cytoplasm and nucleus of transfected cells. The standard curve used for quantitation is shown. The fold change in copy number of viral DNA in p7- and p8-transfected cells compared with empty vector control cells is shown in bar graphs. (C, F) RT-PCR and western blot for caspase 9 gene expression was also checked post p7/p8 transfection and compared with the expression in empty vector control.

Reactivation of the EBV leads to the initiation of the lytic cycle, and thus, the viral particles are assembled in the cytoplasm and released in the media. P7 and p8 were found to interact with the OriLyt and increase the replication of the OriLyt plasmid; hence, we were interested in investigating whether p7 and p8 can induce viral replication. 293T and Bl41 cells were transformed with EBV B95.8 strain, which has a deletion in the genome at p7 and p8 regions. These transformed cells were transfected with p7, p8, or empty vector. Cell culture supernatant was collected, and the cells were harvested 5 days post-transfection. DNA was isolated from the cytoplasmic fraction and the nuclear fraction of the cells and from the culture supernatant. Real-time PCR was performed for the estimation of the copy number of the EBNA1. Standard curve was prepared with a known copy number of EBNA1 (Fig. 7A and D). The fold change in copy number of the viral DNA in p7- and p8-transfected cells were compared based on the empty vector as control. There is a significant increase in the copy number of EBNA1 for both the cell in the supernatant and in the cytoplasmic fraction (Fig. 7B and E), indicating the increase in viral copies. In viral reactivation, the viral particles are formed in the cytoplasm and are subsequently released in the supernatant and probably therefore we also found no significant change of viral copies in the nucleus in both the cells (Fig. 7B and E). These data clearly point out the involvement of p7 and p8 in viral reactivation and the subsequent increase in the viral DNA copy number.

This study shows that EBV non-coding RNAs p7 and p8 promote viral replication by interacting with OriLyt, increasing viral DNA copy numbers in both the cytoplasm and cell culture supernatant. Transfection experiments in EBV-transformed 293T and BL41 cells reveal a significant increase in EBNA1 copies when p7 and p8 are expressed, supporting their role in EBV reactivation. Notably, no change was observed in nuclear viral copies, suggesting that p7 and p8 specifically enhance cytoplasmic replication and release.

### Induction of proliferation and inhibition of host cell apoptosis by p7 and p8

**Fig 8:**
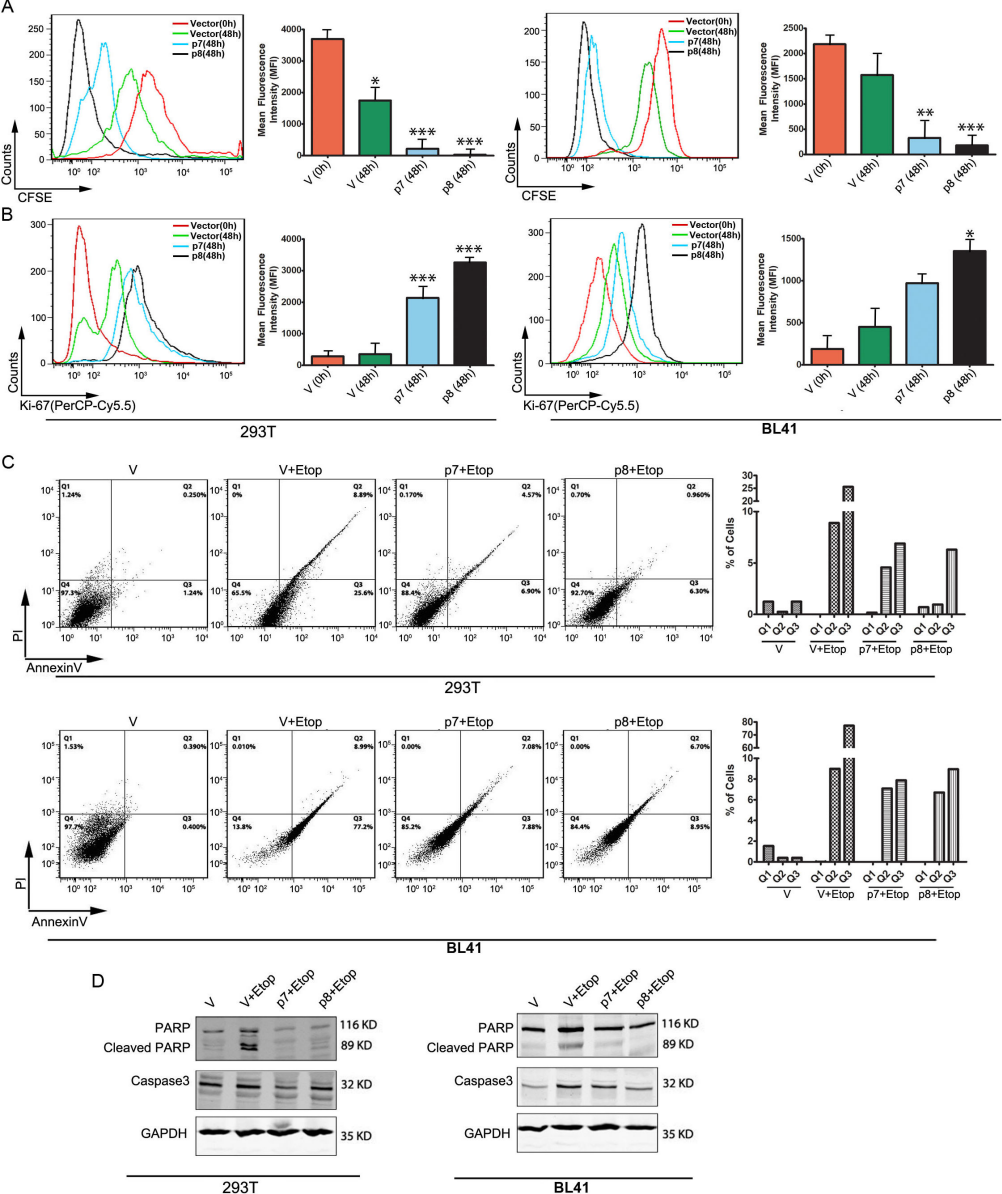
The p7 and p8 RNAs aid in cell proliferation and inhibit apoptosis. (A, B) Hek293T and BL41 cells were transfected with either p7 or p8 or empty vector and incubated with CFSE for 48 h before being fixed for assessed for CFSE stain by flow cytometer. (B) Hek293T and BL41 cells were transfected with either p7 or p8 or empty vector, incubated for 48 h, and stained with fluorescently tagged anti-ki67 antibody. The cells were washed and analyzed using flow cytometry. (C, D) Hek293T and BL41 cells transfected with p7 or p8 were treated with etoposide (10 μM) for 24 h and were either stained with FITC-conjugated annexin-V and propidium iodide or harvested, and western blot was performed to check the status for cleaved PARP and caspase 3.

From CHART analysis, we found other targets that center around p53 and have the potential to influence host cell apoptosis and survival. To confirm whether p7 and p8 have any impact on cellular proliferation, we have transfected p7, p8, or empty vector in BL41 or 293T cells and checked cellular proliferation by CFSE staining and elucidated the levels of Ki67. Flow cytometric analysis clearly revealed that 48 h post-transfection, levels of Ki67 increased considerably (Fig. 8C and D). There is also a significant decrease in the levels of CFSE staining in both cell lines (Fig. 8A and B). The alterations were evident from the graphical presentation of the mean fluorescence intensity (MFI). All these data clearly point toward enhanced cellular proliferation in p7- and p8-transfected cells compared with the vector control.

Induction of apoptosis is characterized by the exposure of phosphatidyl serine in the outer membrane followed by increased cellular permeability. As p7 and p8 were found to interact with caspase 9 and were found to inhibit its expression in both RNA and protein levels (Fig. 7C and F), we were interested to investigate whether p7 and p8 can inhibit the apoptotic signals. 293T and BJAB cells were transfected with p7 or p8 or empty vector and 24 h post-transfection, cells were treated with etoposide (50 μM). Flow cytometric analysis revealed that etoposide increased the % of apoptotic cells, which was restored back in the p7 and p8 transfected samples (Fig. 8E and F). To further confirm these results, the different pro-apoptotic markers like cleavage of PARP, levels of caspase 3 were tested. Etoposide being a positive inducer of apoptosis induced PARP cleavage and caspase 3 and expression. P7 and p8 were found to downregulate both PARP cleavage and caspase expression. These results clearly suggest that p7 and p8 are not only inducing proliferation of the cells but also inhibiting apoptosis.

In conclusion, p7 and p8 enhance cellular proliferation and suppress apoptosis in transfected cells. Increased Ki67 levels and decreased CFSE staining confirm the proliferative effects of p7 and p8, whereas their interaction with caspase 9 and suppression of apoptotic markers like PARP cleavage and caspase 3 further indicate an anti-apoptotic role. Together, these findings demonstrate that p7 and p8 contribute to cell survival by promoting growth and inhibiting apoptosis.

## DISCUSSION

EBV uses diverse strategies to enable long-term persistence within the host, and the expression of different ncRNAs is one of them. EBV ncRNAs and their contributions to viral pathogenesis and oncogenesis have been the subject of intense research since they were first identified. Many primary signaling pathways are now known to be regulated by EBV ncRNAs, such as the NF-κB (28) and Wnt pathways (7), and processes such as apoptosis (29) and immune activation (16). There are more than 40 microRNAs of EBV discovered to date that somehow facilitate EBV for infecting or persisting within the host cells (28).

In the present study using small RNA cloning and sequencing, we have characterized two novel EBV-encoded ncRNAs, namely p7 and p8 (100 nucleotides each), in EBV-infected cell lines. These ncRNAs were identified to be upregulated during lytic reactivation (Fig. 1C) and were found to interact with several regions of host and viral genomes (Fig. 3C). The expressions of both p7 and p8 were also validated in another EBV-positive clonally selected cell line LCL2 and in reactivated nasopharyngeal carcinoma cells (NPCs) transformed by M81 strain of EBV as evident from the real-time data. EBV-negative BJAB cells were taken as negative control. In all these cell lines, namely LCL1, LCL2, and in NPC infected with EBV, the expression levels of p7 and p8 increased from 3 to 5 days post-infection. Expression of BZLF1 was also checked by western blot to ensure proper reactivation. Because of the higher rate of replication of the M81 strain in NPCs, we did detect more p7 and p8 ncRNA transcripts even in the latent cells. p7 and p8 were also found to be mainly localized in the nuclear fraction compared with that of the cytoplasmic fraction.

To elucidate the biological activity of p7 and p8, we performed CHART analysis. Four biotinylated-specific probes against different regions of each of p7 and p8 were designed (Fig. 3A), and their hybridization with p7 and p8 was tested by RNase H assay, which revealed that p7-3 and p8-3 were the most efficient with the highest RNase H sensitivity (Fig. 3B). These two probes were used to pull down the probable targets of p7 and p8. RNA and DNA sequencing and mass spectroscopic analysis were performed for the pulldowns to reveal the interacting partners. p7 and p8 were found to interact with 5 and 7 cellular RNA transcripts, respectively. To check whether the binding of p7 and p8 with these cellular transcripts has any effect on its mRNA expression, we performed rtPCR analysis for these genes. Among all these RNA transcripts, p7 and p8 both were found to decrease the transcript level of ARMCX3, whereas only p8 has a profound effect on PTPN6 and RPL24 (Fig. 4B). Although p7 and p8 were found to have no regulatory control on the mRNA expression of the other RNA transcripts.

EBV miRNAs have been proposed to directly target viral transcripts, including LMP1, to control their levels. BART miRNAs are thought to modulate the expression of LMP1 to maintain a balance between viral latency and active lytic replication (17). Specifically, BART22 miRNA has been suggested to potentially target LMP1, reducing its expression and thus helping EBV evade host immune responses (30). By downregulating LMP1, the virus may limit the host’s immune surveillance, allowing reactivated cells to persist and complete the viral reactivation process. From the CHART analysis, it is clearly evident that p7 has a strong binding affinity to LMP1 (viral transcript); hence, we were interested in determining whether the expression of LMP1 is influenced by p7 or p8. When 293T-EBV or Bl41-B95.8 or 293T-LMP-GFP cells were transfected with plasmids containing p7 or p8, the expression of LMP1 was measured in mRNA as well as protein levels. Both p7 and p8 substantially downregulated the LMP1 expression in both mRNA and protein levels (Fig. 5). IGV alignment analysis clearly reveals that p8 has a strong binding probability with that of LMP1, but interestingly, p7 does not show any significant binding probability. Therefore, the alteration of the mRNA or protein levels of LMP1 on p7 transfection is clearly not by direct interaction of p7 with LMP1 RNA but may be through any other interacting partners or may indirectly influence the expression by modulating some other cellular pathways, which needs further attention.

Among the cellular targets, both p7 and p8 were found to inhibit ARMCX3 (Fig. 4B). ARMCX3 is reported to alter mitochondrial dynamics (31) and is related to induction of inflammation (32). Similarly, p8 was also found to significantly hinder PTPN6 and RPL24 expressions with impairment of PTPN6 being associated with inflammation (33), whereas RPL24 is associated with microRNA processing (34). These findings clearly indicate that both p7 and p8 alter different cellular processes including inflammation, apoptosis, and RNA processing.

The CHART followed by DNA sequencing results revealed that p7 and p8 interact with the OriLyt region of the viral genome. PCR of the CHART pulldown DNAs confirmed that mainly p7 interacts with the OriLyt region of the viral genome. To confirm the CHART results and test the functionality of the p7 and p8 interaction with OriLyt sequence, the replication assay was performed, and p7- and p8-transfected cells have a relatively higher copy number of OriLyt plasmids within the cell post-reactivation compared with the mock-transfected cells, suggesting a positive regulation of viral DNA replication by p7 and p8 RNA. We further reconfirmed these results in 293T and Bl41 cells infected with B95.8 EBV strains (lacking p7 and p8) and calculated the viral copy number in the supernatant, cytoplasmic, and nuclear fractions post-complete reactivation. There is a significant increase in the viral copy number, with p8 showing more predominance over p7.

The expressions of both p7 and p8 were also detectable during latency (Fig. 1C) mainly in cytoplasm but were increased by 12 h post-induction (data not shown) and consistently increased up to 5 days (Fig. 2B). At this time, they were found to be more concentrated in the nucleus with concomitant expression of BZLF1 in the cell lines examined. From this current study, we were not able to ascertain the interconnection between BZLF1 and p7 and p8 expressions. However, BZLF1, p7, and p8 were coexpressed in cells undergoing lytic replication during reactivation. From these data, we hypothesize that both p7 and p8 have nuclear functions during reactivation.

From IPA analysis, we predicted that both p7 and p8 have a direct correlation with cell survival and proliferation through modulation of the p53 pathway. We performed a cell proliferation assay with 293T cells labeled with CFSE dye and transfected with p7 or p8 or mock vector, and 48 h post-transfection, both cell types showed a significant increase in cell proliferation as evident from decreased fluorescence intensity. This finding was reconfirmed by looking into the status of another proliferative marker Ki67 (Fig. 8A through D).

We next shifted our focus to whether p7 and p8 can influence the apoptosis induction in the host cells. P7 and p8 pre-transfected cells were treated with apoptosis, inducing etoposide, and different parameters of apoptosis were studied. Both p7 and p8 were found to inhibit apoptosis induction in the host cells. The levels of proapoptotic proteins also confirmed our previous finding, suggesting that p7 and p8 not only promote the proliferation of the infected host cell but also inhibit the apoptosis induction of the cells.

EBV-encoded non-coding RNAs, including BART miRNAs, EBERs, and lncRNAs, play significant roles in regulating the switch between latency and lytic reactivation (7). Although BART miRNAs primarily suppress reactivation by targeting key viral lytic genes, EBERs support cell survival and immune evasion, creating a favorable environment for the virus during both latency and reactivation (35). The downregulation of BART miRNAs and modulation of EBERs and lncRNAs during reactivation facilitates the viral replication process, allowing EBV to exit latency and enter the lytic cycle (36). Our current research also indicates the existence of other EBV-encoded ncRNA (p7 and p8) that plays a significant role in the reactivation process. We clearly demonstrated that these two ncRNAs inhibit apoptosis induction in the early stage of reactivation, thus giving enough time and facilitating the production of more viral copies.

## MATERIALS AND METHODS

### Cell lines

HEK-293 was maintained in Dulbecco’s modified Eagle’s medium (DMEM; Gibco) supplemented with 10% bovine growth serum (BGS; HyClone), 25 U/mL penicillin, 50 µg/mL streptomycin, and 2 mM L-glutamine. HEK293T cells were transfected with 5–10 µg of BAC GFP-EBV DNA by lipofectamine 2000 (Invitrogen, Inc., Carlsbad, CA) according to the manufacturer’s instruction. After selection with puromycin, the stable puromycin-resistant clones were selected, and the presence of the EBV genome was confirmed by qPCR. EBV-transformed immortalized LCL1 and 2 (lymphoblastoid cell line) cells were generated in our laboratory (37); BL41 and BL41-B95.8 cell lines were kindly provided by Elliott Kieff (Harvard Medical School, Boston, MA), which were grown in Roswell Park Memorial Institute (RPMI 1640) media (Hyclone, Logan, UT) supplemented with 10% FBS, 25 U/mL penicillin, 50 µg/mL streptomycin, and 2 mM L-glutamine. The EBV-positive cells were reactivated using 20 ng/mL 12-O-tetradecanoylphorbol-13-acetate (TPA) and 10 µM of butyric acid.

### Western blot

Cell lysates were electrophoresed on SDS-PAGE gels and transferred to 0.45 µm nitrocellulose membranes. Blots were then probed using specific primary antibodies (S12) for LMP-1 (38) and mouse monoclonal antibodies for caspase 9, PARP1, caspase 3, and GAPDH were purchased from Santa Cruz Inc., Dallas, TX. This was followed by incubation with IR-labeled secondary antibodies, diluted at 1∶20,000. Blots were visualized and analyzed using the LICOR Odyssey imaging system and Odyssey software (Li-Cor, Lincoln, NE).

### Transfection

BL41 cells were transfected by electroporation with a Bio-Rad Gene Pulser II electroporator. Ten million BL41 cells in the exponential phase were harvested by centrifugation at 900 rpm for 5 min. The pellet was washed once with 10 mL phosphate-buffered saline (PBS) and resuspended with 0.4 mL cold RPMI medium without serum, mixed with plasmids and cells. The mixture was transferred into a 0.4 cm cuvette and placed on ice for 5 min. Electroporation was performed at 220 V and 975 µF. After electroporation, the cuvettes were placed on ice for another 10 min, and the cells were transferred into a pre-warmed RPMI medium and incubated at 37°C. Twenty-four hours post-transfection, the transfection efficiency was monitored.

HEK293T cells were grown in a 100 mm cell culture dish at 40%–60% confluency and transfected with 20 µg of the plasmids by calcium phosphate method.

### RNA isolation and real-time PCR

Ten million cells were harvested, and the total RNA was extracted by using a Trizol reagent (Invitrogen, Inc., Carlsbad, CA). Then, cDNA was generated using the Superscript II reverse transcriptase kit (Invitrogen, Inc., Carlsbad, CA) according to the manufacturer’s protocol. Quantitative real-time PCR analysis was performed by using SYBR green real-time master mix (MJ Research Inc., Waltham, MA). The results were normalized to the endogenous control, GAPDH. The list of primers was tabulated in Table S2.

### RNase H sensitivity assay

One hundred million cells were harvested and washed twice with 1× PBS and were crosslinked using 1% formaldehyde. The cell pellet was washed with 1× ice-cold PBS and lysed for 10 min using lysis buffer (10 mM Tris [pH 8], 0.32 M sucrose, 0.1 M EDTA, 3 mM calcium chloride, 0.1% NP40 protease inhibitor cocktail, and 200U of RNase inhibitor). The supernatant was discarded, and the nuclei containing the pellet was washed with wash buffer and sonicated twice for 2 min (15 s on/off cycle) and centrifuged at 16,000 × *g* for 10 min. The clear supernatant was collected and treated with RNase H (5 U), and the specific probes at 30˚C for 30 min. The tubes were added with DNase and incubated at 55˚C for 1 h followed by 65˚C for another 1 h. 90 µl of diethyl pyrocarbonate (DEPC) water were added, and RNA was extracted using Trizol. The total RNA yield was measured by nanodrop and reverse transcribed, and real-time PCR was performed for p7 and p8 using U2 snRNA control.

### Capture hybridization analysis of RNA targets (CHART)

CHART enrichment is performed using formaldehyde‐cross‐linked chromatin extracts that covalently link the RNA with their interacting targets including other cellular RNAs, DNAs, and proteins. For CHART, RNAs were isolated using a similar protocol as that of RNase H sensitivity assay. The interacting partners were finally pulled down for either sequencing or mass-spectroscopic analysis as described previously (23).

### RNA sequencing and analysis

Total RNA was collected from the pulldown, and the quality of the extracted RNA was assessed using an Agilent Bio-Analyzer system (Agilent Technologies, Santa Clara, CA). Sequencing libraries were prepared using the TruSeq stranded mRNA kit (Illumina Inc., San Diego, CA) according to the manufacturer’s protocol. RNA-seq data were collected using an Illumina HiSeq3000 platform in a single-read 50  bp sequencing module at the Genome Sequencing Facility core (Washington University in St. Louis, MO). All samples analyzed passed quality control with the following parameters of the same length (36  bp), 100% coverage in all bases, 25% of A, T, G, and C nucleotide contributions, and 50% GC on base content and less than 0.1% over-represented sequences, indicating good quality. Quality check was performed on the raw RNA-seq reads (FastQC, Site URL), then mapped with the EBV reference genome (the EBV reference genome and annotation were downloaded from https://www.ncbi.nlm.nih.gov/nuccore/NC_007605.1) and hg38 genome (HISAT2).

### Cell proliferation assay

One million HEK293T or BL41 cells were transfected with either p7 or p8 or empty vector, and the cell proliferation was measured after 48 h by Carboxyfluorescein succinimidyl ester (CFSE) as described previously (39) or by the levels of expression of Ki67, which is also a marker for proliferating cells (40).

### Quantification of apoptotic cell death

Fluorescein isothiocyanate (FITC)-conjugated annexin V counterstained with propidium iodide was used to detect apoptosis. Cells transfected with either p7, p8, or empty vector were treated with 10 μM of etoposide for 24 h and stained with FITC-coupled annexin V and PI as described previously (41). The stained cells were analyzed using a BD LSR Fortessa cell analyzer.

## Data Availability

The data presented in the study are deposited in the SAR repository (BioProject ID: PRJNA1217362).

## SUPPLEMENTAL MATERIAL

Captions mbio.04060-24-s0001.docx Supplemental table captions.

Table S1 mbio.04060-24-s0002.docx Probe sequences and their binding affinity for p7 and p8 as determined from PITA software.

Table S2 mbio.04060-24-s0003.docx Primer and probe sequences.

Table S3 mbio.04060-24-s0004.xlsx Cellular and viral DNA and RNA targets of p7 and p8.

ASM does not own the copyrights to Supplemental Material that may be linked to, or accessed through, an article. The authors have granted ASM a non-exclusive, world-wide license to publish the Supplemental Material files. Please contact the corresponding author directly for reuse.
