# Supplementary material for: Novel small non-coding RNAs of Epstein-Barr virus upregulated upon lytic reactivation aid in viral genomic replication and virion production
Source: mBio. 2025 Apr 8;16(5):e04060-24. doi: 10.1128/mbio.04060-24 (PMC12077129; doi:10.1128/mbio.04060-24)
Supplement: Captions — Supplemental table captions. [file mbio.04060-24-s0001.docx]

**Supplementary Table Legends**

**Supplementary Table S1**

Sequences of the probes with their binding position and affinity and Scores that were designed for CHART pull down. The bibdibg affinity and scores were calculated using PITA software (<https://tools4mirs.org/software/target_prediction/pita/>).

**Supplementary Table S2**

Primer and Probe sequences used for rtPCR and pulldown assays.

**Supplementary Table S3**

Cellular and viral DNA and RNA targets with their positions in the genome that coprecipitated with p7 and p8 specific probes.
