# Supplementary material for: Novel small non-coding RNAs of Epstein-Barr virus upregulated upon lytic reactivation aid in viral genomic replication and virion production
Source: mBio. 2025 Apr 8;16(5):e04060-24. doi: 10.1128/mbio.04060-24 (PMC12077129; doi:10.1128/mbio.04060-24)
Supplement: Table S1 — Probe sequences and their binding affinity for p7 and p8 as determined from PITA software. [file mbio.04060-24-s0002.docx]

Supplementary Table S1

For p7 (5’-3’)

Probe p7-1: CATTTTGCACCAGGCCGGGGGAGGTA

Probe p7-2: GTTAGCTTGGATTAGCTGTTAGT

Probe p7-3: TATCAGTTTTCAGCCCGTTTACTGAT

(Through PITA SOFTWARE Probe 3 showed greatest probability of binding)

| ncRNA | Probes | Position | Sites | Seed | dGduplex | dGopen | ddG |
| --- | --- | --- | --- | --- | --- | --- | --- |
| p7 | p7-1 | 18 | 1 | 8:0:0 | -54.2 | -5.65 | -48.54 |
| p7 | p7-2 | 54 | 1 | 8:0:0 | -36 | -6.04 | -29.95 |
| p7 | p7-3 | 85 | 1 | 8:0:0 | -37.5 | -8.53 | -28.96 |

| ncRNA | microRNA | Sites | Score |
| --- | --- | --- | --- |
| p7 | p7-1 | 1 | -48.54 |
| p7 | p7-2 | 1 | -29.95 |
| p7 | p7-3 | 1 | -28.96 |

For p8: 5’-3’

P8-1: TTCTTAGGTGGTGTGTGTTTACAGGGATTAT

P8-2: AGGAGCTCCGGTAGGACCTA

P8-3: GTTAACGATGCCTTGTTCTTAGGTGG

PITA Results:

| ncRNA | Probes | Position | Seed | dGduplex | dGopen | ddG |
| --- | --- | --- | --- | --- | --- | --- |
| p8 | p8-1 | 68 | 8:0:0 | -54 | -2.02 | -51.97 |
| p8 | p8-3 | 83 | 8:0:0 | -44.5 | -10.12 | -34.37 |
| p8 | p8-2 | 30 | 8:0:0 | -42 | -11.61 | -30.38 |
| p8 | p8-2 | 20 | 6:1:0 | -16.4 | -10.93 | -5.46 |
| p8 | p8-3 | 43 | 7:1:1 | -13.1 | -9.74 | -3.35 |
| p8 | p8-3 | 54 | 6:1:1 | -7.44 | -6.65 | -0.78 |
| p8 | p8-3 | 41 | 6:1:1 | -10.02 | -9.74 | -0.27 |

| ncRNA | Probes | Sites | Score |
| --- | --- | --- | --- |
| p8 | p8-1 | 1 | -51.97 |
| p8 | p8-3 | 4 | -34.37 |
| p8 | p8-2 | 2 | -30.38 |
