# Supplementary material for: Novel small non-coding RNAs of Epstein-Barr virus upregulated upon lytic reactivation aid in viral genomic replication and virion production
Source: mBio. 2025 Apr 8;16(5):e04060-24. doi: 10.1128/mbio.04060-24 (PMC12077129; doi:10.1128/mbio.04060-24)
Supplement: Table S2 — Primer and probe sequences. [file mbio.04060-24-s0003.docx]

Supplementary Table 2.

| Primers | 5´- 3´ |
| --- | --- |
| stem loop AS for p8miRNA | GTCGTATCCAGTGCAGGGTCCGAGGTATTCGCACTGGATACGACCAAAG |
| p8miRNA FP | GCA CGC AAC AAG GCA TCG |
| p8miRNA RP | CCA GTG CAG GGT CCG AGG TA |
| Caspase-9 FP | AAG AGT GGC TCC TGG TAC GTT G |
| Caspase-9 RP | TTT CAC CGA AAC AGC ATT AGC G |
| EZH2-FP | AAA GCA CAG TGC AAC ACC AAG C |
| EZH2-RP | TCA GAT GGT GCC AGC AAT AGA TG |
| PCGF4-FP | TAT GCA GCT CAT CCT TCT GCT G |
| PCGF4-RP | TTT CCG ATC CAA TCT GTT CTG GTC |
| APAF1-FP | TCA CTG CAG ATT TTC ACC AGA |
| APAF1-RP | CCT CTC ATT TGC TGA TGT CG |
| E2F6-FP | CTT GAC TTA AAC AAG GTT GCA ACG |
| E2F6-RP | AGC ATC TTC CAT TGC TGA TAA GTC |
| Probes for RNaseH Assay |  |
| Probe p7-1 | CATTTTGCACCAGGCCGGGGGAGGTA |
| Probe p7-2 | GTTAGCTTGGATTAGCTGTTAGT |
| Probe p7-3 | TATCAGTTTTCAGCCCGTTTACTGAT |
| Probe p8-1 | TTCTTAGGTGGTGTGTGTTTACAGGGATTAT |
| Probe p8-2 | AGGAGCTCCGGTAGGACCTA |
| Probe p8-3 | GTTAACGATGCCTTGTTCTTAGGTGG |
| Probe for p7 and p8 RNA CHART |  |
| Antisense p8-3 CHART | CCA CCT AAG AAC AAG GCA TCG TTA AC/iSp18//3BioTEG/ |
| p8-3 CHART | GTT AAC GAT GCC TTG TTC TTA GGT GG/iSp18//3BioTEG/ |
| Antisense p7-3 CHART | ATC AGT AAA CGG GCT GAA AAC TGA TA/iSp18//3BioTEG/ |
| p7-3 CHART | TAT CAG TTT TCA GCC CGT TTA CTG AT/iSp18//3BioTEG/ |
